# Development of an embedded diagnostic tool for visual misalignment screening

**DOI:** 10.1016/j.ohx.2025.e00692

**Published:** 2025-08-19

**Authors:** Daniel Soto Rodriguez, Andres Eduardo Rivera Gomez, Ruthber Rodriguez Serrezuela

**Affiliations:** a Facultad de ingeniería, ingeniería de software, universidad surcolombiana, Neiva, Huila, Colombia; b Master en inteligencia artificial, Universidad de la Rioja, Logroño, España; c Facultad de ingeniería, ingeniería mecatrónica, Corporación universitaria del huila “corhuila”, Neiva, Huila, Colombia

**Keywords:** Strabismus detection, Medical AI, Convolutional neural networks (CNN), Low-cost diagnostic device

## Abstract

This article presents the design, implementation, and validation of a low-cost embedded system for preliminary strabismus screening, based on computer vision and deep learning. The hardware integrates a Raspberry Pi 4, a USB camera, and a 3D-printed chin rest to ensure consistent facial positioning. The software, developed in Python using PyQt5 and OpenCV, incorporates a NASNetLarge convolutional neural network converted to TensorFlow Lite for real-time inference. The graphical interface allows users to capture or upload images, perform automated analysis, generate diagnostic PDF reports, and access a gamified treatment module. Functional validation included a proprietary dataset of 27 images, achieving a 96.30 % classification accuracy. Additionally, a stratified 10-fold cross-validation on a balanced dataset of 1000 images yielded an average accuracy of 95.6 % with strong generalization metrics (F1-score, precision, and recall above 94 %). A novel treatment validation mechanism was implemented by analyzing pupil-to-stimulus distance frame-by-frame, confirming reliable eye tracking and the system’s potential for detecting microstrabismus. This open-source, portable prototype is suitable for community health screening and educational use, particularly in low-resource settings.

## Graphical abstract

**Figure:**
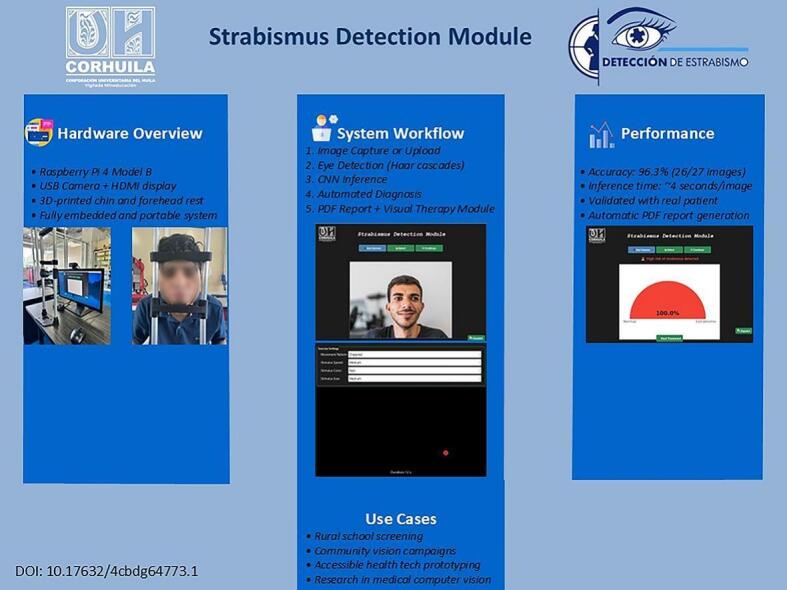

## 1 Hardware in context

**Specifications table.**

**Table:** 

Hardware name	*Strabismus Detection Module*
Subject area	• *Medical (e.g., medical diagnosis, clinical tools, healthcare AI)*
Hardware type	• *Imaging tools* • *Electrical engineering and computer science*
Closest commercial analog	*There is no commercially available equivalent*
Open-source license	*GNU GPL v3.0 (software); CERN-OHL-S 2.0 (electronic designs, CAD files, and 3D printable models)*
Cost of hardware	*USD 259,57*
Source file repository	*Doi Mendeley data:**https://doi.org/10.17632/4cbdg64773.1*

*Strabismus is a prevalent ophthalmological condition in the pediatric population, affecting between 2 % and 4 % of young children and representing one of the primary causes of irreversible amblyopia if it is not detected and treated in a timely manner* [1], [2]. *Amblyopia, resulting from the cortical suppression of the visual signal originating from the deviated eye, constitutes a critical condition, as once it has developed, it cannot be reversed through conventional methods such as eyeglasses or surgery* [3], [4]*. This situation makes the development of low-cost, automated, and objective solutions imperative for the early detection of strabismus, particularly in regions with limited access to ophthalmology specialists* [5], [6]*.*

*Traditionally, the diagnosis of strabismus has been performed using clinical methods such as the Hirschberg test and the cover test, which, although widely used, are subject to the clinician’s experience and influenced by factors such as patient cooperation, lighting conditions, and anatomical variability* [7]*. Given these limitations, various studies have proposed the use of image processing techniques and deep learning as complementary tools for the automated diagnosis of ocular pathologies.* [8], [9]*.*

*In response to this need, a low-cost embedded prototype has been developed for the preliminary automated detection of strabismus, employing computer vision and artificial intelligence. The system has been fully implemented on a Raspberry Pi 4 Model B, equipped with a generic USB camera for facial image acquisition and an HDMI display for interactive result visualization. This setup enables real-time analysis of ocular alignment based on pre-trained convolutional neural networks (CNNs). The software was developed in Python using PyQt5 for the graphical user interface, OpenCV for image acquisition and processing, and TensorFlow with an optimized model in TensorFlow Lite format for efficient execution on the Raspberry Pi’s ARMv8 architecture. The model used is derived from NASNetLarge, fine-tuned for binary classification (normal vs. strabismus), and trained with clinically validated, pre-segmented facial images. The system features a user-friendly interface that allows the operator to capture real-time images or load local images, automatically detect the ocular region, preprocess the image, and generate an automatic diagnosis accompanied by a semicircular gauge-style graphical visualization. Additionally, in cases where strabismus is detected, the system activates a gamified visual rehabilitation module designed to guide the patient through personalized ocular fixation exercises.*

*The physical integration includes a 3D-printed stand with a chin rest to stabilize the patient’s face during image capture, thereby improving the model’s accuracy by standardizing the acquisition angle. Due to its portability, low power consumption, and ease of use, this prototype represents a viable alternative for early visual screening in school settings, community eye health campaigns, and regions with limited access to specialists.*

*Compared to other approaches based on EOG signals or complex eye-tracking systems* [10]*, this solution stands out for its simplicity, operational autonomy, and ease of replication, without the need for specialized biomedical hardware. Its modular and open design positions it as an accessible diagnostic device, expandable and adaptable to lighter or more accurate AI architectures in future iterations.*

## 2 Hardware description

*The developed device is a fully embedded preliminary strabismus detection system, built from low-cost and open-source components. It is designed to be used without specialized clinical intervention, facilitating its adoption in health campaigns, schools, or rural centers. Unlike commercial ophthalmological equipment, this hardware:*

*It does not require specialized lenses, infrared-based eye tracking, or facial markers, nor does it employ invasive biomedical signals (such as EOG or EEG), thereby reducing both cost and complexity. It can be operated by non-specialized technical personnel thanks to its intuitive graphical interface.*

*The system consists of a Raspberry Pi 4 Model B, a generic USB camera, and an HDMI display, all mounted on a custom physical base fabricated using 3D printing. This design includes an adjustable frontal chin rest, which ensures facial stability during image capture. The internal architecture is optimized to run a convolutional model in TensorFlow Lite, previously trained on facial images with and without strabismus.*

*Instead of applying advanced image processing techniques, the system relies on optimized image capture conditions to ensure effective detection of key facial structures (eyes and pupils). The use of good-quality USB cameras connected to a Raspberry Pi device is recommended, along with proper lighting, stable framing, and sharp focus. These conditions enable the implemented classic algorithms—such as Haar-based detection and contour analysis—to operate reliably and efficiently. This design choice supports a lightweight, adaptable, and practical solution suitable for real-world deployment on low-power embedded systems. Furthermore, to ensure accurate eye detection and pupil tracking, it is recommended to maintain a distance of approximately 30 to 50 cm, not exceeding 50 cm, between the user's eye and the camera. This range allows the full face to be captured with sufficient detail for the detection algorithms (Haar classifiers and contour segmentation) to function properly, while minimizing blur and improving lighting without requiring advanced processing techniques.*

*Compared to conventional ophthalmological hardware, this system:*

*It reduces the implementation cost to less than 5 % of the value of a clinical diagnostic device, requires no advanced calibration or specific laboratory conditions (e.g., controlled lighting), and operates autonomously and portably, powered by a standard 5 V source.*

*This prototype not only provides preliminary diagnosis but also includes a gamified visual treatment module, based on personalized exercises that respond to the detected degree of deviation.*

### 2.1 Applicability and transfer potential

*This hardware may be useful for researchers or developers of health, education, or clinical assistance technologies in various contexts. The following are potential applications:*

*Visual neuroscience: as an experimental platform for evaluating responses to controlled visual stimuli without the need for professional eye-tracking equipment.*

*Community clinics: for mass visual screening in rural schools or areas without access to ophthalmologists.*

*Accessible interface development: as a foundation for creating adaptive interfaces for individuals with ocular dysfunction.*

*Computer vision in low-resource settings: for investigating the detection of ocular facial features in environments without GPUs or specialized cameras.*

## 3 Design files summary

**Table:** 

***File or folder name***	***File type***	***Licencia open source***	***File location***
*Software*	*Software package*	*GNU GPL v3.0*	Source file repository
*Electronic Design/esquematico.fzz*	*Electronic diagram (Fritzing)*	*CERN-OHL-S v2.0*	Source file repository
Chin Rest Design/*.SLDPRT	*CAD files of individual parts (SolidWorks)*	*CERN-OHL-S v2.0*	Source file repository
*Chin Rest Design/Ensamble.SLDASM*	*Complete 3D assembly (SolidWorks)*	*CERN-OHL-S v2.0*	Source file repository
stl/*.stl	*Files for 3D printing*	*CERN-OHL-S v2.0*	Source file repository

### 3.1 Description of the design files

• ***Software:*** *The system features a graphical user interface developed in Python, specifically designed for execution on Raspberry Pi OS. While the interface is provided as a precompiled Debian installer (estrabism1.0.deb) to facilitate deployment, the full source code is also included in the public repository, in compliance with the principles of free and open-source software and the GNU GPL v3.0 license.*

The **/Software**/ directory contains the following components:

• ***estrabism1.0.deb****: the compiled installer package for Debian-based systems.*
• ***/src/:*** *the full source code of the application in Python, organized as follows:*

*1.main.py* *: the main script that controls the system’s logic and user interface.*

*2.translations.py* *: contains the multilingual dictionary (LANGUAGES) supporting both Spanish and English interfaces, enabling users to switch languages dynamically during runtime.*

*3.* ***Clasificadores/*** *: includes Haar cascade classifiers (haarcascade_frontalface_default.xml, haarcascade_eye.xml) for facial and eye detection.*

*4.* ***Modelos/*** *: stores the trained machine learning model in. tflite format used for strabismus prediction.*

*5.****icons/, Logos/, and gif****/: directories containing visual assets such as UI icons, institutional branding, and loading* animations*.*

• ***Electronic design/esquematico.fzz:*** *Schematic generated in Fritzing detailing the system connections between the Raspberry Pi, the USB camera, the HDMI display, and the power supply.*
• Chin rest design/*.SLDPRT*: Individual parts designed in SolidWorks, including support bars, base platform, and anatomical models of the human head.*
• ***Chin rest design /Ensamble.SLDASM****: Complete assembly of the physical system that positions and stabilizes the patient's head in front of the camera. It facilitates replication in clinical settings.*
• stl/*.stl*: 3D printing-ready files exported from CAD designs. They enable precise and low-cost fabrication of the physical support system in PLA using FDM printers.*

## 4 Bill of materials summary

**Table:** 

***Designator***	***Component***	***Quantity***	***Unit cost (USD)***	***Total cost (USD)***	***Source***	***Material Type***
*RPI4B*	*Raspberry Pi 4 Model B (4 GB RAM)*	*1*	*81,15*	*81,15*	*sigmaelectronica.net*	*Semiconductor*
*VENPI4*	*Raspberry Pi 3 fan 5v Reprap Prusa I3 30x30x10mm*	*1*	*4,06*	*4,06*	*Mercadolibre.com.co*	*Electronics*
*CAMUSB*	*Generic HD USB camera*	*1*	*31,74*	*31,74*	*Mercadolibre.com.co*	*Electronics*
*HDMI7LCD*	*22″ HDMI display*	*1*	*108,14*	*108,14*	*Mercadolibre.com.co*	*Electronics*
*FDMPLA01*	*PLA filament (1 kg)*	*0.2*	*21,16*	*21,16*	*Arrow 3D*	*Polymer*
*PWRUSB5V3A*	*5 V 3A power adapter*	*1*	*9,17*	*9,17*	*Mercadolibre.com.co*	*Electronics*
*SD32GB*	*32 GB microSD card*	*1*	*6,77*	*6,77*	*Mercadolibre.com.co*	*Electronics*
*STLHOLDER*	*3D-printed components (chin rest)*	*4*	*4,23*	*4,23*	*Included STL files (self-fabrication)*	*Polymer (PLA)*

## 5 Build instructions

*The construction of the system is divided into three main blocks: physical assembly of the chin rest, connection of the processing module (Raspberry Pi 4), and deployment of the strabismus analysis software.*

### 5.1 Chin rest assembly

• *3D prints the four parts provided in the attached STL files using PLA filament or preferably ABS, with a layer height of 0.2 mm, an infill percentage of 80 %, and support enabled to enhance structural stability.*
• *The assembly sequence of the printed parts follows the following order (from bottom to top):*

1 Lower base (part 4).

2 Chin support (part 2).

3 Adjustable forehead support (part 3).

4 Upper reinforcement (part 1).

• *The parts are designed to be connected using structural tubes (to be added later), which pass through the assembly holes to provide longitudinal rigidity. No glue or screws are required for the current printed components.*
• *It is recommended to verify that the dimensions of the tubes match the insertion diameters of the components.*

### 5.2 Connection of the raspberry Pi and peripherals

• *The Raspberry Pi 4 Model B is connected independently and is not physically integrated into the support system.*
• *An HDMI display is connected through the HDMI port of the Raspberry Pi.*
• *A generic USB camera is connected directly to one of the USB ports of the Raspberry Pi.*
• *The power supply is provided through two sources:*

1 5 V-3A power supply for the Raspberry Pi.

2 12 V power supply (if required for the display, depending on the model).

• *A cooling fan is installed and connected to the 5 V and GND GPIO pins of the Raspberry Pi, in accordance with the provided electrical schematic (see attached figures).*
• *All connections are standard and don’t require soldering* (see Fig. 2)*.*

### 5.3 Software installation

• *Install the Raspberry Pi OS using the official Raspberry Pi Imager tool. Follow the instructions provided in Getting Started with Your Raspberry Pi.*
• *Open a terminal on the Raspberry Pi and run the following commands:*

cd /home/pi.

sudo dpkg –i estrabism1.0.deb.

If any dependency errors appear, resolve them by executing:

sudo apt-get install –f.

This will automatically install any missing system dependency such as python3-venv.

• *What happens during installation:*

The application files are copied to:

/opt/estrabismo/.

A Python virtual environment is created in:

/opt/estrabismo/env/.

1.All required Python libraries are installed within that virtual environment using pip.

2.A command-line launcher named estrabismo is registered.

3.A desktop shortcut is created and added to the system’s application menu.

• *To launch the application:*

Simply type the following command in the terminal:

estrabismo.

Or launch it via the Applications Menu, under the “Education”.

### 5.4 Safety considerations

*Ensure proper ventilation of the Raspberry Pi to prevent overheating, as continuous execution of graphical interfaces in PyQt5 and AI inference processing imposes a high load on the CPU and RAM. Handle the 3D-printed components with care, avoiding drops or exposure to excessive moisture that could compromise the material’s integrity. Use original 5 V-3A chargers to avoid electrical overloads.*

## 6 Operation instructions

*The strabismus detection module operates by capturing facial images, automatically processing them through convolutional neural networks, and generating a preliminary diagnosis displayed via a user-friendly graphical interface. The detailed operating instructions are as follows:*

### 6.1 System startup

• *Turn on the Raspberry Pi and the connected HDMI display.*
• *Access the graphical environment of Raspberry Pi OS.*
• *Locate and launch the shortcut created in the application menu (Strabismus Detection).*

### 6.2 Graphical interface usage

• *Upon launching the program, a main window will appear with three primary buttons:*

7 Use Camera: opens a real-time capture window to take a photograph of the patient’s face or eyes.

8 Select Image: allows the user to load a previously stored image from local memory.

9 Continue: analyzes the loaded or captured image to perform the diagnosis.

*Real-time image capture:*

• *Select the Use Camera option.*
• *Adjust the patient's position in front of the camera using the chin rest system.*
• *Define the capture mode (full face or eyes only) using the mode switch button.*
• *Capture the image once the framing is appropriate.*

*Local image upload:*

• *Select the Select Image option.*
• *Navigate to the desired image in.jpg,.jpeg, or.png format* (see Fig. 3).

### 6.3 Performing the analysis

• *Once an image has been loaded or captured, press the Continue button.*
• *The patient’s basic information (name and age) will be requested through an input form.*
• *The system will automatically detect the ocular region and process the image using the TensorFlow Lite model (tflite).*
• *A semicircular gauge-style chart will be displayed, indicating the probability of strabismus, accompanied by an interpretive textual diagnosis.*
• *Additionally, a PDF report containing the results, analyzed image, and prediction graph will be generated and saved in the reportes/ folder* (see Fig. 4).

### 6.4 Visual treatment

• *If strabismus is detected (probability greater than 50 %), a Start Treatment button will be enabled.*
• *The treatment consists of gamified visual exercises in which the patient must follow a moving stimulus with their gaze. The stimulus is configurable in terms of motion pattern, speed, size, and color.*
• *The treatment module records the duration of the exercise performed by the patient.*
• *The visual treatment window includes a button labeled* ***“Start Tracking”****, which activates the real-time gaze tracking system. Once pressed, the system begins to monitor the patient's eye movement relative to the stimulus. This feature enables accurate detection of gaze alignment or deviation throughout the treatment session, supporting the identification of subtle oculomotor irregularities* (see Fig. 5)*.*

### 6.5 Change interface language

*The language can be switched between Spanish and English using the English/español button located in the bottom right corner.*

### 6.6 Safety considerations during operation

• *Do not power off the Raspberry Pi abruptly to avoid damage to the file system.*
• *Ensure proper ventilation of the Raspberry Pi by using an active cooling fan.*
• *Avoid exposing electronic devices to moisture or high temperatures.*
• *Supervise patients during image capture or visual exercises to prevent sudden movements or accidental falls.*

## 7 Validation and characterization

### 7.1 Operation demonstration

*The functional validation of the prototype was conducted using a proprietary dataset composed of 27 images not included in the training set: 26 images corresponding to patients with confirmed strabismus and 1 control image without strabismus. Although the validation set was intentionally imbalanced toward positive cases, it was primarily employed to verify the correct operation of the entire workflow (capture → analysis → report generation → treatment activation). The optimized NASNetLarge model (NASNETLARGE.tflite), executed on Raspberry Pi 4, correctly classified 26 out of 27 images, achieving an overall accuracy of 96.30 %.*

*Additionally, real-time capture tests were conducted with a clinically diagnosed strabismus patient, yielding a positive prediction with a probability of 100 %, successfully activating the visual treatment module. These preliminary results confirm the functional robustness of the system; however, future studies will include larger and more balanced validation datasets to enable a comprehensive statistical analysis of clinical performance.*

### 7.2 Relevant use case

*The system is intended for strabismus screening in the general population, with no age restrictions, provided the user can follow basic ocular fixation instructions.*

*The total processing time—from image selection or capture to diagnosis display—is approximately 4 s per image.*

### 7.3 Characterization parameters

**Fig. 1:**
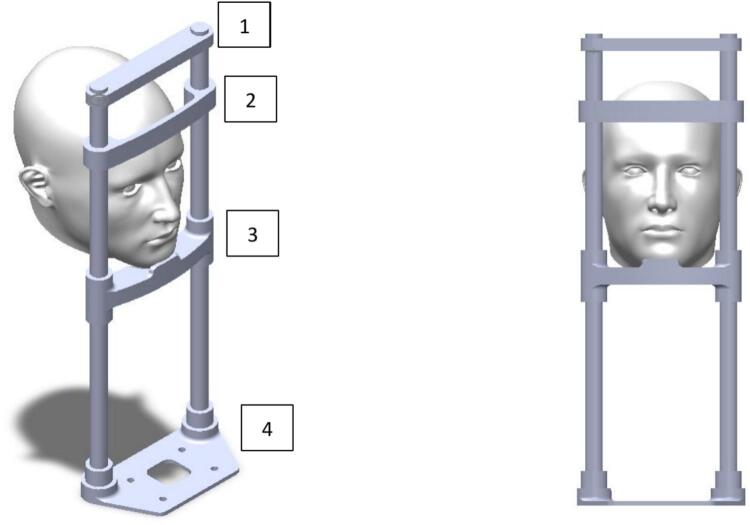
Chin rest design, 1. Lower base, 2. Chin support, 3. Adjustable forehead support, 4. Upper reinforcement.

• *System-level classification accuracy: 96.30 % during functional validation with 27 new images (26 positive, 1 negative) using the deployed prototype. This evaluation was intended to verify end-to-end performance in a real-world scenario.*
• *Average inference time: ∼4 s per image on Raspberry Pi 4.*
• *The system operates with a frame acquisition rate of 1 frame per second (1 FPS) during the diagnostic process, which has proven sufficient for performing accurate predictions using a clear, static image—consistent with the deep learning model's training data. This choice enables lightweight processing, suitable for low-cost devices or educational environments. Nevertheless, the system’s modular architecture allows future adaptation to higher frame rates (e.g., 5–10 FPS), aiming to enhance the detection of subtle or transient ocular deviations in more demanding clinical settings.*
• *Operational stability: The system remained stable during continuous usage sessions of up to 1 h, with no crashes or severe overheating.*
• *Automatic report generation: Each diagnosis produces a PDF report including the analyzed image, the prediction percentage, and the interpretive diagnosis* Fig. 1.

### 7.4 Statistical evaluation and model reliability.

*To validate the robustness of the proposed classification model, a 10-fold stratified cross-validation approach was applied to a balanced dataset comprising 500 images of patients with strabismus and 500 images of normal subjects. Stratification ensured that each training and validation subset preserved the class distribution, which is essential for avoiding bias in models trained on clinical datasets* Fig. 6.

**Fig. 2:**
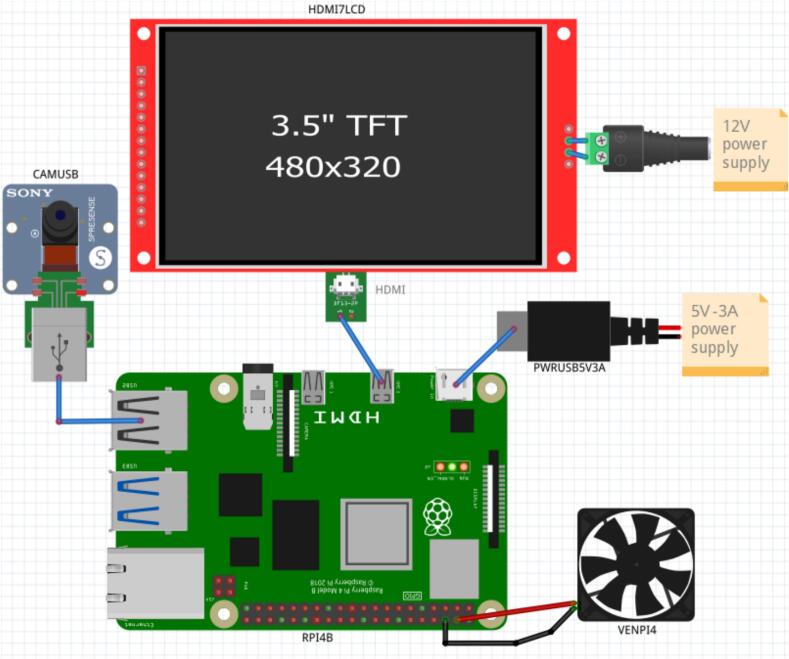
Schematic design.

**Fig. 3:**
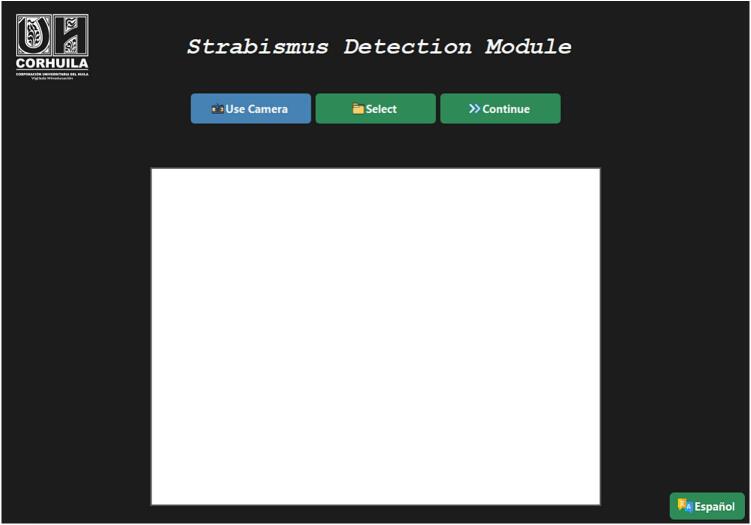
Main program window.

**Fig. 4:**
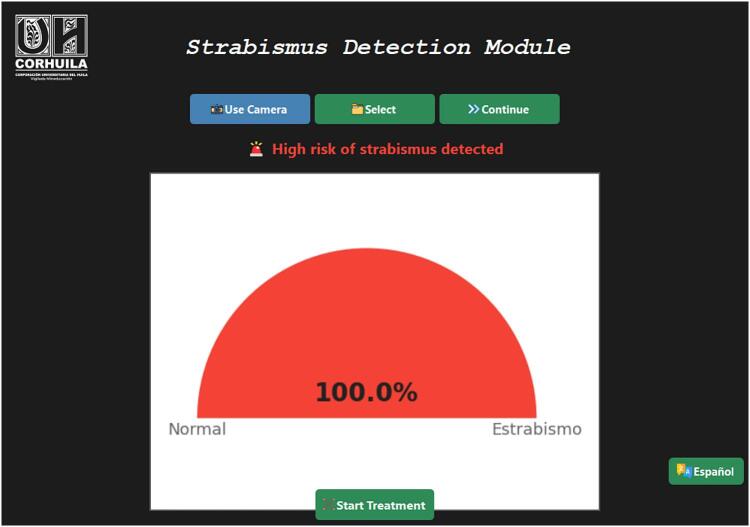
Captured Image Analysis Window.

**Fig. 5:**
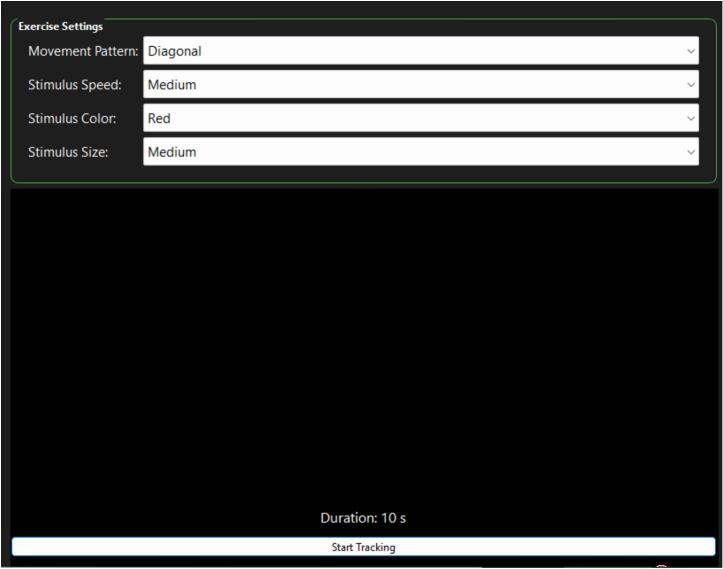
Treatment window.

**Fig. 6:**
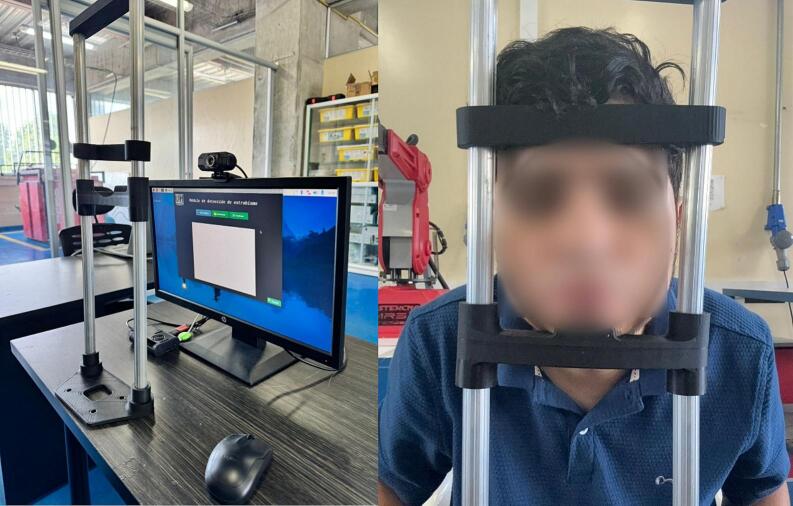
Strabismus Detection Module.

*During each iteration of the validation process, performance metrics such as accuracy, precision, recall, and F1-score were calculated for both classes (* Table 1 *). These values were collected per fold and are presented in detail in a table showing the individual results. Additionally, a statistical summary was computed for each metric, including the mean, standard deviation, and 95 % confidence interval (CI 95 %) (* Table 2 *).*

**Table 1:** Stratified 10-fold cross-validation results by fold.

***Fold***	***Accuracy***	***Val_Accuracy***	***Strabismus Precision***	***Strabismus Recall***	***Strabismus F1-score***	***Normal Precision***	***Normal Recall***	***Normal F1-score***
*1*	*0.9523*	*0.9423*	*0.9259*	*0.9615*	*0.9434*	*0.96*	*0.9231*	*0.9412*
*2*	*0.99*	*0.98*	*1*	*0.9615*	*0.9804*	*0.963*	*1*	*0.9811*
*3*	*0.9715*	*0.9615*	*0.9286*	*1*	*0.963*	*1*	*0.9231*	*0.96*
*4*	*0.9523*	*0.9423*	*0.9259*	*0.9615*	*0.9434*	*0.96*	*0.9231*	*0.9412*
*5*	*0.9331*	*0.9231*	*0.9583*	*0.8846*	*0.92*	*0.8929*	*0.9615*	*0.9259*
*6*	*0.9331*	*0.9231*	*0.9231*	*0.9231*	*0.9231*	*0.9231*	*0.9231*	*0.9231*
*7*	*0.9331*	*0.9231*	*0.8667*	*1*	*0.9286*	*1*	*0.8462*	*0.9167*
*8*	*0.9715*	*0.9615*	*1*	*0.9231*	*0.96*	*0.9286*	*1*	*0.963*
*9*	*0.9523*	*0.9423*	*0.9259*	*0.9615*	*0.9434*	*0.96*	*0.9231*	*0.9412*
*10*	*0.9715*	*0.9615*	*1*	*0.9231*	*0.96*	*0.9286*	*1*	*0.963*

**Table 2:** Summary statistics (mean, standard deviation, and 95% confidence intervals) across folds.

	***Mean***	***Std***	***95 % CI***
*Accuracy final*	*0.9561*	*±0.0197*	*0.0122*
*Val_Accuracy final*	*0.9461*	*±0.0197*	*0.0122*
*Strabismus Precision*	*0.9454*	*±0.0437*	*0.0271*
*Strabismus Recall*	*0.95*	*±0.0365*	*0.0226*
*Strabismus F1-score*	*0.9465*	*±0.0194*	*0.0120*
*Normal Precision*	*0.9516*	*±0.0339*	*0.0210*
*Normal Recall*	*0.9423*	*±0.0488*	*0.0302*
*Normal F1-score*	*0.9456*	*±0.0207*	*0.0128*

*This interval was estimated using the following formula:*

(1) $$\begin{array}{c} \mathrm{IC}=1.96x\left(\frac{\sigma }{\sqrt{n}}\right) \end{array}$$

*where IC represents the 95 % confidence interval (expressed as a percentage, %), σ denotes the standard deviation of the evaluated metric (%, dimensionless ratio when expressed in decimal form), and n is the total number of folds used in the stratified cross-validation (dimensionless; in this study, n = 10).*

*This evaluation strategy enables a more accurate assessment of the model’s generalization capacity, even in contexts with limited data. Although the dataset size is not massive, the use of data augmentation techniques during training—such as rotations, translations, brightness adjustments, and zoom—helped enrich the representation of visual variations and mitigate potential overfitting effects.*

*The results obtained show an average classification accuracy above 94 % and a balanced performance between both classes, suggesting that the model has good discriminative capability. This statistical validation is essential to support the system’s reliability and directly addresses the reviewers’ observations regarding the need for greater rigor in the model’s quantitative evaluation.*

**Fig. 7:**
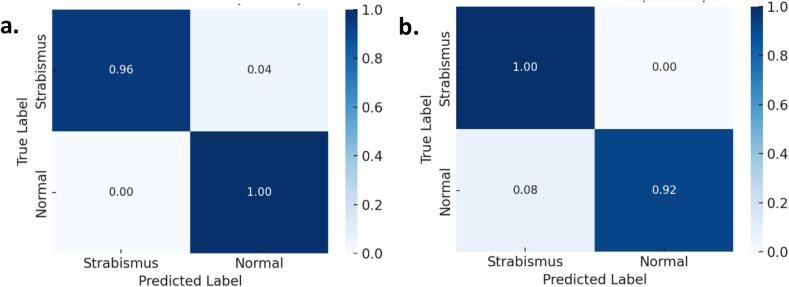
Normalized confusion matrices: (a) Fold 2 and (b) Fold 3 of the stratified 10-fold cross-validation.

*To complement the quantitative analysis of the trained model,* Fig. 7 *presents the normalized confusion matrices corresponding to Folds 2 and 3, which achieved the highest validation accuracy during the stratified cross-validation process. These matrices were normalized by row, allowing visualization of the relative proportion of correct and incorrect predictions for each class (strabismus and normal).*

*As evidenced in* Fig. 7*, the model achieved perfect precision for the 'strabismus' class and near-perfect recall for both classes, demonstrating a high capacity to correctly detect strabismus cases while minimizing false positives. Similarly, Fold 3 exhibits a consistent classification pattern, with perfect recall in the 'strabismus' class and high precision in the 'normal' class.*

*These results reinforce the robustness of the model within the context of stratified cross-validation and confirm its effectiveness in distinguishing between both categories, even when trained on a moderately limited dataset enhanced through data augmentation techniques.*

### 7.5 Validation of ocular tracking mechanism during treatment sessions.

*To validate the proper functioning of the visual treatment module, a real-time comparison mechanism was developed between the expected trajectory of the stimulus and the estimated movement of the user's pupil. Upon initiating the treatment session, the user selects the exercise parameters (motion pattern, color, size, and speed), which are recorded as reference values for gaze-tracking analysis.*

**Fig. 8:**
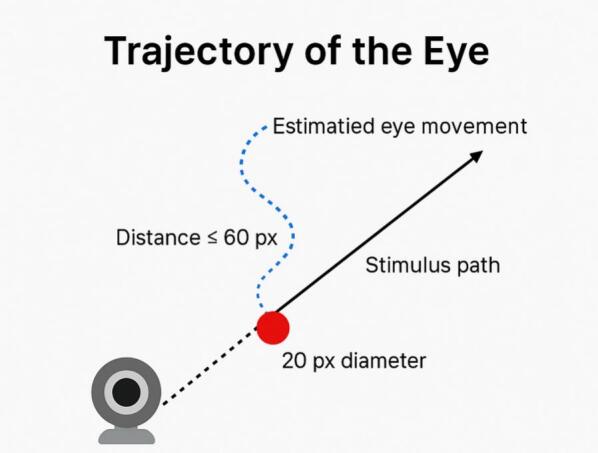
Reference-Based Eye Tracking Mechanism during Visual Stimulus Movement.

*During execution, the system calculates the Euclidean distance between the center of the stimulus and the detected pupil position in each frame. If this distance remains below a predefined threshold (60 pixels), the stimulus is correctly followed.* Fig. 8 *presents a schematic representation of this validation process, illustrating the stimulus path (in red) and the estimated eye movement within the acceptable tracking range.*

**Fig. 9:**
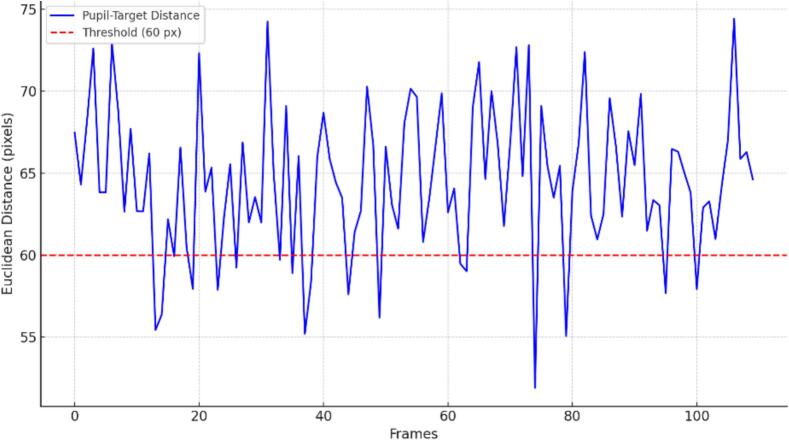
Frame-by-Frame Analysis of Pupil–Stimulus Distance.

*Additionally, for each session, the software automatically generates a graph of pupil-to-stimulus distance over time, as shown in* Fig. 9*. This visualization allows for an assessment of tracking performance throughout the session. The red dashed line represents the 60-pixel threshold and facilitates the identification of isolated deviations or patterns of unstable fixation. This approach provides both quantitative and visual evidence to validate whether the patient's eye is adequately following the stimulus, and it may assist in detecting subtle oculomotor anomalies such as microstrabismus or inconsistent ocular control*.

### 7.6 Validation of the recommended camera-to-face distance

*In the prototype, a recommended operational distance between the user’s face and the USB camera was empirically set to 30–50 cm. To rigorously justify this range, we applied a geometric analysis based on the* ***horizontal field-of-view equation derived from the pinhole camera model****, a standard formulation in computer vision and optical engineering* [11]*. The goal was to ensure that the face and, in particular, the eyes occupy sufficient pixel resolution in the captured image for accurate detection and classification.*

*The width of the visible area captured by the camera at a given distance D is estimated by:*

(2) $$\begin{array}{c} W=2*D*\tan\left(\frac{\theta }{2}\right) \end{array}$$

*where W is the horizontal width of the visible area captured by the camera (in centimeters, cm), D is the distance from the camera to the subject’s face (cm), and θ is the horizontal field of view of the camera lens (in radians; 60° ≈ 1.047 rad for typical webcam optics).*

*Assuming* $\theta =60^{{}^{\circ}}$*, typical of webcam lenses, the horizontal capture width at 30 cm is:*

$\begin{array}{c} W=2\cdot 0.3\cdot tan\left(30{}^{\circ}\right)\approx 34.6cm \end{array}$ *(3)nd at 50 cm:*

(4) $$\begin{array}{c} W=2\cdot 0.5\cdot tan\left(30{}^{\circ}\right)\approx 57.7cm \end{array}$$

*iven that the average human face is approximately 16 cm wide, this ensures the entire face is well framed at both limits. Moreover, considering a 1920-pixel-wide image, the effective resolution at 30 cm corresponds to ∼0.22 mm/pixel, allowing the eyes and pupils (typically 1–1.2 cm wide) to occupy 20–30 pixels, which is sufficient for accurate detection by Haar cascades and convolutional networks.*

**Fig. 10:**
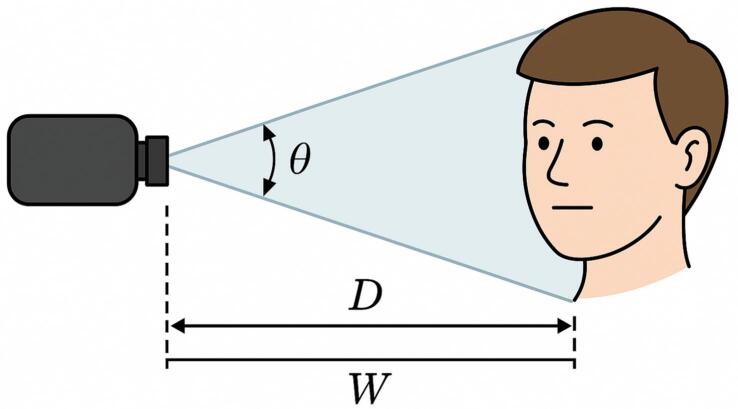
Conceptual illustration of the horizontal field of view (FOV) of a USB camera. Note: Dimensions not to scale. W is calculated using the pinhole camera model: W = 2D⋅tan(θ/2) author.

*Distances beyond 50 cm reduce eye region size below acceptable thresholds, while closer distances risk blurring due to fixed-focus optics. Thus, the 30–50 cm range is not arbitrary, but results from a balanced trade-off between field of view, resolution, and optical sharpness* (see Fig. 10).

### 7.7 Structural validation of the chin rest support

*To ensure the mechanical reliability of the chin support structure integrated into the prototype for strabismus detection, a Finite Element Analysis (FEA) was conducted using static loading conditions. The goal was to verify that the support could withstand the operational forces exerted by users during use, while maintaining structural integrity and minimal deformation to preserve camera alignment and facial positioning accuracy.*

*The chin support was subjected to a combination of loads based on expected user interaction. The upper contact zone (forehead rest) received a load of* ***1 kgf****, represented in the simulations by* ***red arrows****, simulating the gentle resting force of the user's head. The chin platform, being the primary load-bearing area, was subjected to a force of* ***20 kgf****, indicated by* ***brown or orange arrows****, representing the vertical force exerted by the user's chin under normal conditions.*

**Fig. 11:**
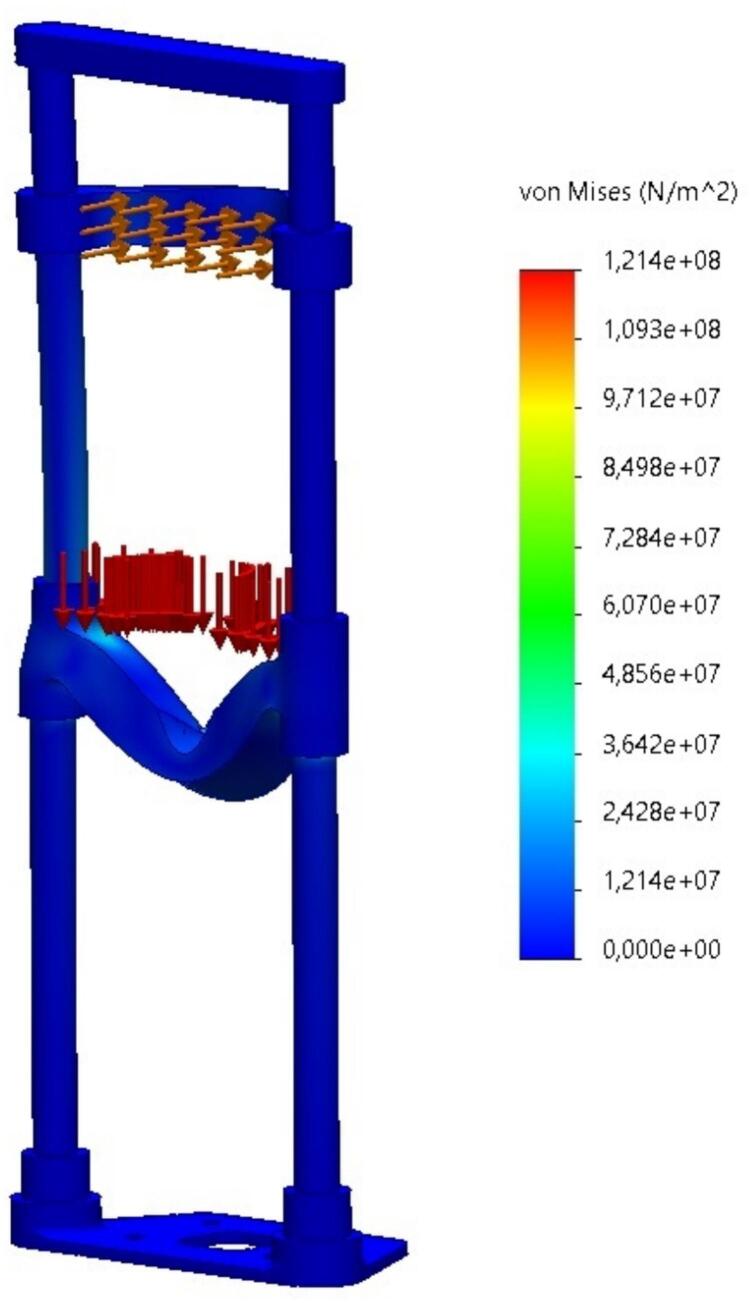
Finite element simulation showing the von Mises stress distribution in the chin support structure. The highest stress occurs near the curvature of the chin platform, author.

Fig. 11 *illustrates the* ***von Mises stress distribution*** *throughout the structure. The stress is concentrated around the curvature of the chin platform, with peak values well below the material’s yield strength, confirming the safety margin of the design*.

**Fig. 12:**
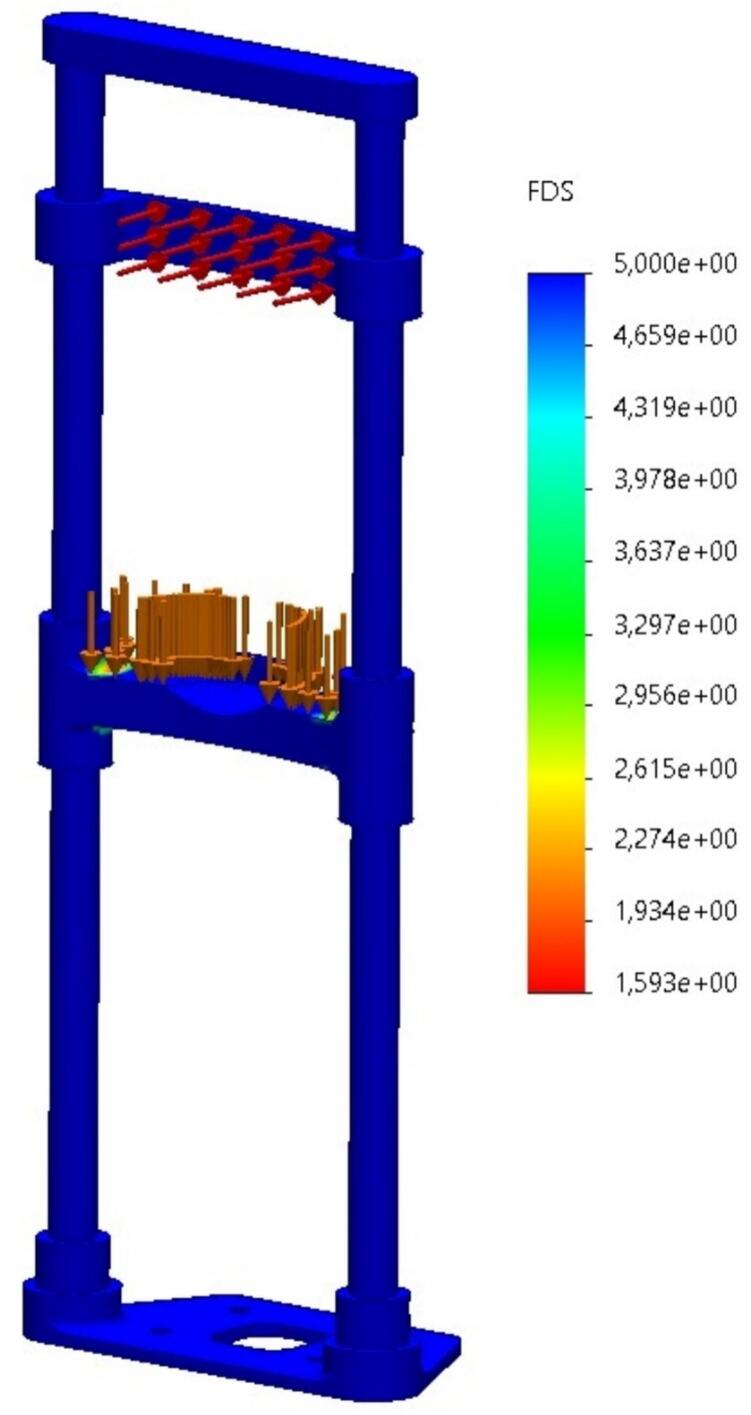
Factor of Safety (FOS) analysis of the prototype. The minimum FOS is 1.6, ensuring structural integrity under operational conditions, author.

*As shown in* Fig. 12*, the* ***Factor of Safety (FOS)*** *analysis indicates a minimum FOS of* ***1.6****, which is acceptable for non-critical, low-impact medical devices. This suggests that even under exaggerated loading conditions, the structure remains within a safe operating range.*

**Fig. 13:**
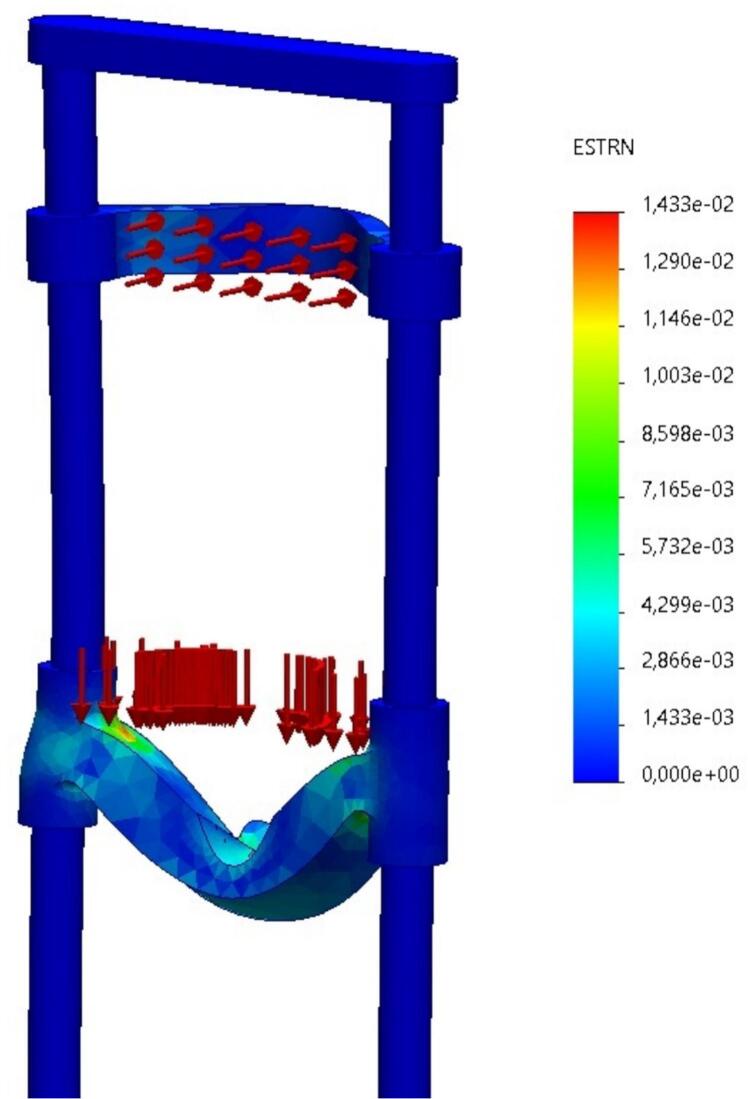
Unit strain distribution across the structure, confirming elastic behavior of the chin platform under applied loads, author.

*The* ***unit strain distribution****, depicted in* Fig. 13*, demonstrates that the material remains in the elastic range, with no signs of plastic deformation. This confirms the resilience of the structure during repeated usage.*

**Fig. 14:**
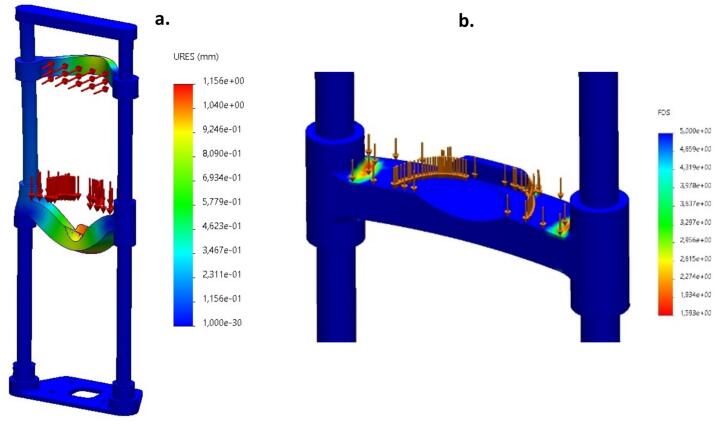
A) Static displacement simulation showing minimal deflection, preserving system alignment and performance. B) Zoomed-in FOS visualization of the chin platform showing localized stress regions. Autor.

Fig. 14***a*** *presents the* ***static displacement field****, showing a maximum displacement of approximately* ***1.15 mm****, localized primarily at the central chin rest region. This value is within acceptable tolerance, ensuring the device retains its functional alignment and accurate face-to-camera positioning.*

*If necessary,* Fig. 14***b*** *provides a zoomed-in analysis of localized safety factors around critical mounting points, validating the robustness of the fastening and support geometry.*

*This FEA-based structural validation confirms that the chin support system is mechanically robust and suitable for the proposed application in a vision-based strabismus detection prototype.*

### 7.8 Hardware capabilities and limitations

#### 7.8.1 Capabilities

• *The system offers low implementation costs, representing an economically accessible alternative to commercial clinical ophthalmological devices.*
• *It features high portability, allowing for easy transport and installation in various clinical or educational settings without the need for specialized technical requirements.*
• *The graphical user interface developed in PyQt5 provides an intuitive and user-friendly operating environment, enabling use by personnel without specialized expertise in information technologies.*
• *The platform enables automatic generation of PDF reports, including the analyzed image, prediction result, and interpretive diagnosis, thereby enhancing clinical traceability.*
• *The system demonstrates the capability to detect subtle ocular deviations, including cases of microstrabismus, from frontal facial images.*
• *It incorporates a gamified visual treatment module, configurable in difficulty parameters (speed, motion pattern, color, and stimulus size), aimed at strengthening ocular coordination.*
• *The application provides multilingual support, allowing dynamic switching between Spanish and English during operation.*

### 7.9 Limitations

• *The system's effectiveness heavily depends on the quality of the camera used; low-resolution cameras or optical deficiencies may negatively impact diagnostic accuracy.*
• *Sensitivity to inadequate lighting conditions may affect the effectiveness of facial and ocular detection due to the reliance on OpenCV Haar classifiers.*
• *The system requires precise frontal alignment of the patient's face for a valid capture; angular deviations may compromise eye detection.*
• *Currently, detection is optimized for frontal images; lateral or tilted captures may not be interpreted correctly.*
• *Advanced image preprocessing techniques, such as histogram normalization, edge enhancement, or automatic white balance, are not implemented, which may limit the robustness of the analysis under suboptimal conditions.*

*Overall, the results from experimental validation, the observed operational stability, and the ease of deployment of the proposed system confirm its feasibility as a preliminary strabismus screening tool in clinical and educational contexts. Although the prototype presents limitations inherent to its architectural simplicity, its modular design, low cost, and automatic detection capabilities position it as an attractive alternative to expand access to early visual assessment in vulnerable populations. Future work will consider the incorporation of image enhancement techniques, expansion of the validation dataset, and optimization of ocular segmentation algorithms to further improve the system’s sensitivity and specificity.*

## Ethics statements

*This study involved the participation of a human subject for the real-time validation of th0065 hardware prototype.*

*The subject voluntarily provided written informed consent for the acquisition, analysis, and publication of non-identifiable images, in accordance with the ethical standards outlined in the Declaration of Helsinki.*

*The signed informed consent form has been made available as* supplementary material *in the public repository associated with this article.*

## CRediT authorship contribution statement

**Daniel Soto Rodriguez:** Investigation. **Andres Eduardo Rivera Gomez:** Investigation. **Ruthber Rodriguez Serrezuela:** Investigation.

## Declaration of competing interest

The authors declare that they have no known competing financial interests or personal relationships that could have appeared to influence the work reported in this paper.
